# Modifications of cellulose-based biomaterials for biomedical applications

**DOI:** 10.3389/fbioe.2022.993711

**Published:** 2022-11-03

**Authors:** Nour Fatema, Ruben Michael Ceballos, Chenguang Fan

**Affiliations:** ^1^ Cell and Molecular Biology Program, University of Arkansas, Fayetteville, AR, United States; ^2^ Department of Biological Sciences, University of Arkansas, Fayetteville, AR, United States; ^3^ Department of Chemistry and Biochemistry, University of Arkansas, Fayetteville, AR, United States

**Keywords:** cellulose, nanocellulose, hydrogel, biomaterial, biomedicine

## Abstract

Cellulose is one of the most abundant organic compounds in nature and is available from diverse sources. Cellulose features tunable properties, making it a promising substrate for biomaterial development. In this review, we highlight advances in the physical processes and chemical modifications of cellulose that enhance its properties for use as a biomaterial. Three cellulosic products are discussed, including nanofibrillated, nanocrystalline, and bacterial cellulose, with a focus on how each may serve as a platform for the development of advanced cellulose-based biomaterials for Biomedical applications. In addition to associating mechanical and chemical properties of cellulosic materials to specific applications, a prospectus is offered for the future development of cellulose-based biomaterials for biomedicine.

## Introduction

Cellulose is the most abundant, broadly-distributed natural polymer in the world (Moon et al., 2011). It is composed of glucose residues linked by β-1,4-glycosidic bonds. Natural fibers from cellulosic feedstock and synthetic cellulose are used in textiles, food, construction, and many other industries (Zhu et al., 2016; Yang et al., 2021). The biocompatibility, biodegradability, water-retention capacity, renewability, and tunability of cellulose make it an ideal biopolymer for use as a biomaterial (Bhaladhare and Das, 2022). In general, cellulosic materials are considered to be environment-friendly and are low-cost when compared to other conventional synthetic materials (Hickey and Pelling, 2019). Cellulose polymers for biomaterials may be produced either by chemical synthesis or biosynthesis. Feedstock from a variety of sources (e.g., plants, animals, and microbes) serve as substrates to produce cellulose-based materials (He et al., 2016; Okeyoshi et al., 2018).

Over the past decade, there has been renewed interest in the use of cellulosic feedstocks to produce biofuels as fuel prices fluctuate erratically and use of fossil fuels continue to contribute to geopolitical instability and climate change (Ceballos, 2017; Kumar et al., 2020; Saravanan et al., 2022). In addition, other research has focused on the physical and chemical properties of cellulose for the development of cellulose-based biomaterials (Habibi, 2014; Agarwal and Csóka, 2019; Tarrahi et al., 2022). It has been shown that cellulose fibers produce elongated fibrillary structures or intact rod-like crystalline particles in the nanoscale range when subjected to mechanical shearing or controlled acid hydrolysis (Klemm et al., 2011). This is advantageous because it permits useful modifications to the macromolecular structure of cellulose (through a variety of chemistries) with nanoscale tunability for a myriad of sophisticated applications (Habibi, 2014).

Several reviews are published on using nanoscale, structured cellulosic substrates (i.e., nanocellulose) in biomaterials. These are mainly focused on sourcing, isolation, fabrication, and surface modifications to cellulose (Hickey and Pelling, 2019; Moohan et al., 2020; Sood et al., 2021; Mali and Sherje, 2022). Although these reports offer details regarding synthesis of cellulosic materials, forming composites, and current applications for cellulosic biomaterials (Agarwal and Csóka, 2019; Tarrahi et al., 2022), few is focused on the compatibility between particular physical processes and chemical properties and the suitability of the resulting nanocellulose-based materials for specific biomedical applications. Here, we aim to connect the features of cellulose-based materials based on their physical and chemical properties to biomedical applications. This review addresses different types of cellulose-based substrates (e.g., nanofibrillated, nanocrystalline, and bacterial cellulose) and the benefits of selected chemical and physical treatments that are amenable for biomedical applications of cellulose-based materials.

## Modifications in the synthesis of cellulose-based biomaterials

**FIGURE 1:**
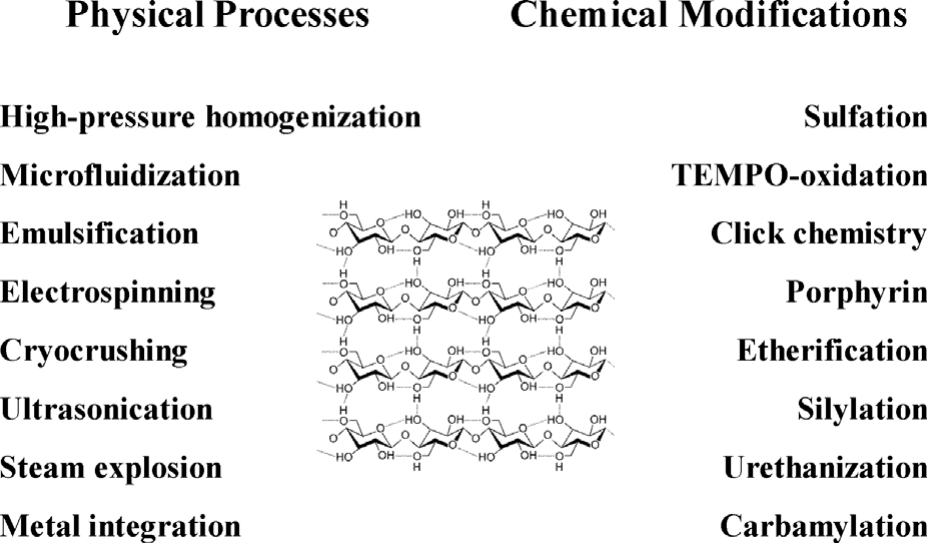
Modifications in the synthesis of cellulose-based biomaterials.

Over the last decade, improvements for the use of cellulose as a biomaterial have included modifying surface properties and constructing cellulose-based composites to serve a wider range of applications (Habibi, 2014; Jorfi and Foster, 2015). Structured cellulose with nanoscale features (i.e., nanocellulose) that include a high aspect (i.e., length-to-width) ratio and a large (micro- to macroscopic) surface area (Agarwal and Csóka, 2019) can be broadly classified as either nanofibrillated cellulose (NFC), nanocrystalline cellulose (NCC), or bacterial cellulose (BC) depending upon its source and properties (Lin and Dufresne, 2014). Functionality of cellulosic materials can be modified surface alterations, including physical adsorption of molecules, attachment of chemical moieties, and derivatization by one or more functional groups (Figure 1).

### Modifications by physical or mechanical processes

Mechanical shearing laterally disintegrates cellulose fibers into sub-structural nanoscale units, called nanofibrils, resulting in nanofibrillated cellulose (NFC) (Habibi, 2014). Rigorous mechanical disruption produces NFC, which features fibrils on the order of several microns (Orlando et al., 2020). Three main technologies, homogenization, microfluidization, and microgrinding, are widely used for mechanical treatment of substrate leading to NFC. For example, a high-pressure homogenization method, which combines a homogenizer and a microfluidizer, is one of the most common substrate treatments due high defibrillation efficiency and relatively short isolation times compared to other methods. During high-pressure homogenization, high shear forces produce defibrillated cellulose fibers from both crystalline and amorphous domains of the cellulose substrate (Kose et al., 2011; Habibi, 2014). Another method to produce NFC is emulsification in which agitation of a multi-phase mixture yields small aqueous droplets of hydrogel precursors in a hydrophobic medium (i.e., oil or organic solvent). This is a proven strategy to produce of nano- or micro-sphere gels (El-Sherbiny and Yacoub, 2013).

NFC may also be produce by other methods including cryocrushing, ultrasonication, and steam explosion (Uetani and Yano, 2011). Cryocrushing involves a combination of severe shearing of cellulose in a refiner, followed by high-impact crushing under liquid nitrogen. Resulting microfibrils are useful in the production of high strength and high stiffness composites for high-performance applications like bone tissue and prosthetics engineering (Chakraborty et al., 2005). In ultrasonication, purified cellulose is soaked in deionized water and then subjected to ultrasonic fibrillation to isolate nanofibers. The process can be performed at a different frequencies and output power levels depending upon the purpose of the process (Xie et al., 2016). Ultrasonication yields nanofibers with desired properties, such as high crystallinity and thermal resistance (Chen et al., 2011), which are used as nanocomposites, filtration media, or films that feature optical transparency (Chakraborty et al., 2005; Chen et al., 2011). Steam explosion is another alternative process for NFC production, in which saturated steam is used to treat cellulosic feedstock. NFC derived from steam explosion exhibits a notable increase in tensile strength as well as improvement in other properties, such as reduced lignin content (Chakraborty et al., 2005).

For BC, silver has been integrated into cellulose by soaking feedstock with various substances, including silver salts (Chen et al., 2019), silver sulfadiazine (Aris et al., 2019), and silver-based fluorescent complexes (deBoer et al., 2015). Other metals such as titanium oxide (Ullah M. W. et al., 2016b), zinc or zinc oxide (Wahid et al., 2019; Dharmalingam and Anandalakshmi, 2020; Dinca et al., 2020), and zeolites or montmorillonite (Horue et al., 2020) have also been integrated into BC biomaterials. BC acts as stabilizing agent to control particle nucleation. Therefore, integration of metal nanoparticles into BC is promising strategy homogeneously incorporating metal nanoparticles and controlling particle formation. In general, the biocompatibility, high specific surface area, and non-toxicity of BC are properties that have prompted rapid development of BC-based biomaterials (Sureshkumar et al., 2010).

### Modifications by chemical alteration

In addition to physical processes, chemical modifications have also been used to develop cellulose-based biomaterials for specific applications. For example, sulfation introduces highly negative sulfate esters on the surface of NCC. This, in turn, can enhance adsorption of select biomolecules such as enzymes (Chen et al., 2013). The 2,2,6,6-tetramethylpiperidine-1-oxyl (TEMPO)-mediated oxidation of cellulose is a widely used method to change the hydroxymethyl groups of cellulose to carboxylic forms while conserving secondary hydroxyls (Besbes et al., 2011; Isogai et al., 2011). Cellulose has also been explored as a substrate for carrying out reactions by click chemistry. Click chemistry produces a group of reactions that are fast, simple to use, easy to purify, versatile, regiospecific, and give high product yields (Hein et al., 2008). For example, porphyrin was covalently immobilized to NCC *via* a 1,3-dipolar cycloaddition catalyzed by Cu(I), which resulted in photodynamic inactivation of *Mycobacterium smegmatis* and *Staphylococcus aureus*. *Escherichia coli* was also inactivated but at lower efficacy (Feese et al., 2011). Etherification has been used as a cost effective and highly efficient chemical treatment step to facilitate the defibrillation of the fibers (Hasani et al., 2008; Eyholzer et al., 2012). Etherification of cellulose by aqueous sodium hydroxide may be followed by cationic surface functionalization of NCC or NFC with ammonium groups *via* the addition of a nucleophile. Surface modifications to NCC or NFC have also been done through silylation with alkyldimethylchlorosilanes followed by isocyanate treatment (Gousse et al., 2002; Andresen et al., 2007). Either NCC or NFC can be treated with isocyanate, which results in urethane linkages *via* urethanization or carbamylation. This enhances the molecular hydrophobicity of the material (Siqueira et al., 2013).

Chemical modification of a cellulose substrate either alone or in conjunction with mechanical or physical treatment may endow the emerging cellulose-based material with a unique set of properties. Selection of manufacturing processes yields biomaterial with desired thermal stability, tensile strength, crystallinity, and other factors. Different material profiles can be matched to compatible applications.

## Modifications of cellulose-based biomaterials for biomedical applications

**FIGURE 2:**
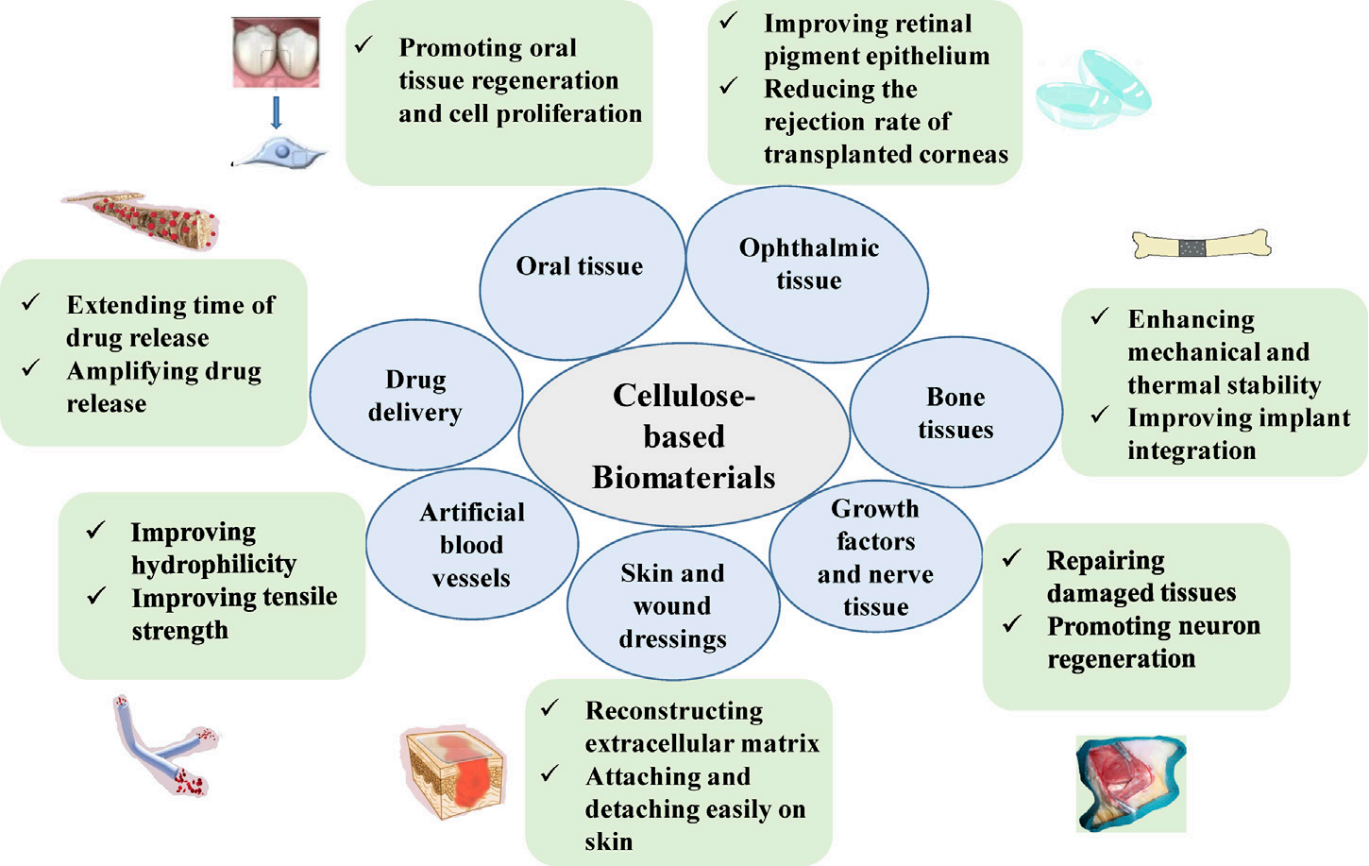
Biomedical applications and advantages of cellulose-based materials.

The use of cellulose as a biomaterial has a long history. Physical processes or chemical modifications of cellulose (Orlando et al., 2020), derivatization of cellulose (Yang et al., 2021), or mixing cellulose with other materials to produce composites (Aris et al., 2019; Wahid et al., 2019) have all resulted in the development of innovative and useful biomaterials. These cellulose-based materials are becoming increasingly useful in biomedicine, including diagnosis, treatment, prevention, and analysis of disease and disease progression (Figure 2).

### Drug delivery

Cellulose and nanocellulose have been used in the form of gels, membranes, spheres, and crystals as excipients for a wide range of drugs (Agarwal and Csóka, 2019). Early literature reports that periodate-oxidized cellulose can be used to immobilize insulin or *p*-amino salicylic acid for prolonged drug delivery (Singh et al., 1981; Bala et al., 1982). In addition, by copolymerizing methacrylic acid, N-isopropyl acrylamide, or ethylene glycol dimethacrylate and employing silica microspheres modified by 3-methacryloxypropyltrimethoxysilane as a template, cellulose-coated hollow microspheres have been engineered to enable prolonged (i.e., slow release) drug delivery (Agarwal and Csóka, 2019). In contrast, for a rapid and controlled drug delivery, oxidized cellulose beads have been developed. Specifically, TEMPO-mediated oxidation provides a pH-responsive system for drug release from beads cellulose beads, which can be tuned to retain drug at pH 1.2 and release drug at pH 7.0 (Xie et al., 2021). The release rate is controlled by changes in oxidation state, allowing drug release at different locations with strategic timing (Xie et al., 2021). Cellulose beads can also be derived from BC. For example, a high-absorbance BC membrane was developed for sustained release of the anti-inflammatory drug diclofenac in transdermal systems (Silva et al., 2014). Using glycerol to facilitate drug absorption and enhance membrane malleability, diclofenac was more readily absorbed (Agarwal and Csóka, 2019). In another report, benzalkonium chloride-treated BC dry films were found to have high drug-loading capacity and enhanced drug efficacy for at least 24 h against *Staphylococcus aureus* and *Bacillus* subtilis when applied to wounds (Ullah H. et al., 2016a).

### Skin and wound dressings

Cellulose-based materials have been used in wound healing to mimic skin, facilitate rapid regeneration of skin cells, and minimize scarring (Hickey and Pelling, 2019). Among the most advanced materials used in wound dressings are those produced *via* bioprinting. Nanocellulose can be an ideal component of bio-ink. For example, nanocellulose fibrils derived from TEMPO-mediated oxidation reduce viscosity in bioprinting yielding advantageous rheological properties (Rees et al., 2015). Bioprinting with nanocellulose-based bio-ink also permits the construction of porous nanostructures to stabilize functionalized molecules. For example, carboxymethylated-periodate nanocellulose has been used in bioprinting to produce 3D porous structures with the capacity to carry and launch microbicides (Rees et al., 2015). Electrospinning is a useful method for the production of 3D porous matrices that mimic the natural structures of layers within skin. Mixtures of cellulose acetate and hydrogel (e.g., gelatin and poly urethane) have been used in electrospinning processes to form scaffolds (Vatankhah et al., 2014). By varying the ratio of nanocellulose-to-hydrogel, parameters such as porosity, stiffness, hydrophobicity, absorption, and surface area can be tuned to improve efficiency in wound healing applications (Liu et al., 2012). The high specific surface area and hydrophilicity of NFC allows it to hold large amounts of water (relative to its dry mass). When dispersed in water, NFC yields a hydrogel that can be modified for a variety of purposes including the production of wound dressing products. It has been shown that functionalized cellulose dressings are superior to existing commercial products such as Suprathel (Hakkarainen et al., 2016).

### Bone tissue engineering

Cellulose has been used in bone tissue engineering because cellulose fibers resemble the collagen fibers of bone tissue and are compatible with the stiff, mechanical environment found in bone systems (Torgbo and Sukyai, 2018; Vallejo et al., 2021). Because the mechanical properties of hydrogels cannot withstand mechanical stresses seen on bone, they are often reinforced with nanocellulose (e.g., NFC). Cellulose nanocrystals (e.g., NCC) serve as support in electrospun matrices of polylactic acid (PLA) or polyvinyl alcohol (PVA) hydrogels (Chalal et al., 2014; Rescignano et al., 2014; Zhang et al., 2015). It has been demonstrated that adhesion between PLA and cellulose in electrospinning can be enhanced by maleic anhydride grafting, polyethylene glycol grafting (PEG), and sodium dodecyl sulfate (SDS). This process modifies the nanocrystals to produce matrices with smaller diameters and polydispersity (Zhou et al., 2013). It also increases mechanical and thermal stability. For example, it has been reported that PLA-cellulose scaffolds can exhibit tensile strengths greater than 10 MPa (Zhang et al., 2015). Fibrous nanocellulose has been used with bioactive glass to coat metal implants resulting in rapid mineralization (e.g., hydroxyapatite formation) to facilitate cell attachment and proliferation around the implants (Chen et al., 2015). Thus, high mechanical strength cellulose and cellulose composites are being successfully implemented in bone tissue regeneration applications.

### Nerve tissue repair and growth factor delivery

Cellulosic materials have been used as scaffolds for nerve cell and stem cell culturing as well as for the delivery of growth factors into tissues of the nervous system (Wang et al., 2013; Du et al., 2014; Kuzmenko et al., 2016). For example, cellulose-based biomaterials have been shown to promote the regeneration of neurons after spinal cord injury. NFC scaffolds are used in research to promote *in vitro* neural stem cell differentiation. *In vivo*, the tunable porosity of NFC scaffolds can facilitate optimal release of growth factors into injured spinal cord regions (Tsai et al., 2006; Hackett et al., 2010). For targeted delivery into micro-environments surrounding neural stem cells, cellulose-based scaffolds have been used to transport and release growth factors. This is useful for heterogeneous neural differentiation of large populations of stem cells and for repairing damaged nerve tissues (Wang et al., 2013).

### Ophthalmic tissue repair

Cellulose-based materials have been developed for several ophthalmic applications. For example, BC/polyvinyl alcohol (PVA) composites are being developed to mimic properties of the natural cornea, which offers a transparent structure with high light transmittance, flexibility but with mechanical strength, and desirable thermal properties (Wang et al., 2010). BC-based contact lens and lens components can be doped with antibiotics, such as ciprofloxacin/γ-cyclodextrin to prevent infection or to treat active bacterial infections (Cavicchioli et al., 2015). BC biomaterials that are modified with chitosan and carboxymethyl cellulose to maximize hydrophilicity have been shown to facilitate enhances propagation of retinal pigment epithelial cells (Goncalves et al., 2015). This offers new prospects in the treatment of multiple eyes diseases including age-related macular degeneration.

### Oral tissue repair

Nanocellulose-based materials have also been developed for oral tissue repair and post-surgical recovery. For example, a blend of BC with calcium chloride and sodium alginate has resulted in the construction of a cellulosic “sponge”. This material has been shown to promote the proliferation of gingival fibroblast cells (Chiaoprakobkij et al., 2011). Similar BC-based materials have shown utility in recovery regimens in root canal surgeries. Specifically, BC biomaterials for plugging cavities from dental root canals showed the ability to expand and cover the entire canal space with the added benefit of sterilizing and removing residue from the canal space (Yoshino et al., 2013).

### Artificial blood vessels

Cellulosic biomaterials have also been used in the regeneration and replacement of vasculature. BC can be molded to very different shapes during its synthesis to generate substrates optimized for enhancing cell attachment and proliferation (Mohite and Patil, 2014; Picheth et al., 2017). Studies have demonstrated that in vascular grafting, materials made with BC induce a reduction in thrombin at target surfaces thus inhibiting clot formation (Fink et al., 2010). This is a notable advantage over other commonly materials commonly used for vascular grafting (e.g., PET and PTFE). In addition, BC-derived composites have emerged as a major alternative in the replacement of atherosclerotic blood vessels. For example, blending BC nanocrystals with PVA (Polyvinyl alcohol) results in an artificial vessel with high tensile strength, low cytotoxicity, and enhanced suture retention profile (Tang et al., 2015). A key issue in implants is optimizing cell adhesion. The development of hydrophilic BC-based biomaterials with polyethylene glycol (PEG) grafted into the cellulosic base have shown favorable compatibility for cell proliferation and adhesion (e.g., fibroblasts), reduced propensity for complement activation, and resistance to bacterial adhesion (da Silva et al., 2016). The development of such hydrophilic BC composites offers notable advances in the development of novel artificial blood vessels implants, coatings for cardiovascular stents (resistant to bacterial adhesion), and the replacement heart valves.

## Prospectus

Development of nanocellulose-based biomaterials is a robust area of current research and engineering. From feedstock choice to defining properties of different cellulosic substrates and matching pretreatment and manufacturing processes to specific applications, the diversity and number of cellulosic biomaterials is growing. In this review, we have summarized general properties of three common cellulosic materials (i.e., NCC, NFC, BC) and discussed physical and chemical processes used to produce or modify each. We have provided examples of how these starting materials are being used in different biomedical applications and why the unique properties of each cellulose substrate are suitable to each application. Due to the sensitivity of biological systems to foreign materials and the tunability of cellulosic substrates, the use of cellulose-based biomaterials for biomedical applications is a robust area of research and development. The shear abundance of cellulose as a raw material and its status as a sustainable resource make cellulosic materials even more attractive. Efforts to understand the limitations of cellulosic biomaterials in biomedicine, such as the potential for immunological rejection, facilitating disease transmission, and enhancing risks for future malignancies are valuable as cellulose-based products become more widely used in biomedicine (Savoji et al., 2018). The potential is great, indeed. Cellulose is being used in bio-ink that serves to produce scaffolds for the regeneration of tissues or entire organs (Weng et al., 2021). Cellulose matrices are being used to stabilize differentiating stem cells and in tissue engineering. Cellulose-based drug delivery systems (e.g., cellulose microspheres and nanobeads) are used to regulate the controlled release of medications and growth factors with high resolution and specificity. Thus, despite some limitations such as production costs for advanced cellulosic substrates (Tornello et al., 2016) and special transportation/storage conditions (Guan et al., 2020), the future looks promising for the use of cellulose-based biomaterials in biomedicine.
